# Bone Metabolite Profile Differs between Normal and Femur Head Necrosis (FHN/BCO)-Affected Broilers: Implications for Dysregulated Metabolic Cascades in FHN Pathophysiology

**DOI:** 10.3390/metabo13050662

**Published:** 2023-05-16

**Authors:** Alison Ramser, Rachel Hawken, Elizabeth Greene, Ron Okimoto, Brenda Flack, Courtney J. Christopher, Shawn R. Campagna, Sami Dridi

**Affiliations:** 1 Center of Excellence for Poultry Science, University of Arkansas, Fayetteville, AR 72701, USA; 2 Cobb-Vantress, Siloam Springs, AR 72761, USA; 3 Department of Chemistry, University of Tennessee, Knoxville, TN 37996, USA; 4 Biological and Small Molecule Mass Spectrometry Core, University of Tennessee at Knoxville, Knoxville, TN 37996, USA

**Keywords:** femur head necrosis, broiler, metabolome, bacterial chondronecrosis with osteomyelitis

## Abstract

Femur head necrosis (FHN), also known as bacterial chondronecrosis with osteomyelitis (BCO), has remained an animal welfare and production concern for modern broilers regardless of efforts to select against it in primary breeder flocks. Characterized by the bacterial infection of weak bone, FHN has been found in birds without clinical lameness and remains only detectable via necropsy. This presents an opportunity to utilize untargeted metabolomics to elucidate potential non-invasive biomarkers and key causative pathways involved in FHN pathology. The current study used ultra-performance liquid chromatography coupled with high-resolution mass spectrometry (UPLC–HRMS) and identified a total of 152 metabolites. Mean intensity differences at *p* < 0.05 were found in 44 metabolites, with 3 significantly down-regulated and 41 up-regulated in FHN-affected bone. Multivariate analysis and a partial least squares discriminant analysis (PLS-DA) scores plot showed the distinct clustering of metabolite profiles from FHN-affected vs. normal bone. Biologically related molecular networks were predicted using an ingenuity pathway analysis (IPA) knowledge base. Using a fold-change cut off of −1.5 and 1.5, top canonical pathways, networks, diseases, molecular functions, and upstream regulators were generated using the 44 differentially abundant metabolites. The results showed the metabolites NAD+, NADP+, and NADH to be downregulated, while 5-Aminoimidazole-4-carboxamide ribonucleotide (AICAR) and histamine were significantly increased in FHN. Ascorbate recycling and purine nucleotides degradation were the top canonical pathways, indicating the potential dysregulation of redox homeostasis and osteogenesis. Lipid metabolism and cellular growth and proliferation were some of the top molecular functions predicted based on the metabolite profile in FHN-affected bone. Network analysis showed significant overlap across metabolites and predicted upstream and downstream complexes, including AMP-activated protein kinase (AMPK), insulin, collagen type IV, mitochondrial complex, c-Jun N-terminal kinase (Jnk), extracellular signal-regulated kinase (ERK), and 3β-hydroxysteroid dehydrogenase (3β HSD). The qPCR analysis of relevant factors showed a significant decrease in *AMPKα2* mRNA expression in FHN-affected bone, supporting the predicted downregulation found in the IPA network analysis. Taken as a whole, these results demonstrate a shift in energy production, bone homeostasis, and bone cell differentiation that is distinct in FHN-affected bone, with implications for how metabolites drive the pathology of FHN.

## 1. Introduction

Femur head necrosis (FHN), also called bacterial chondronecrosis with osteomyelitis (BCO), is a debilitating bacterial infection of weakened bone in growing broiler (meat-type) chickens. The pathology of FHN has been postulated, with the development of a spontaneous experimental model of FHN further supporting the theory [1]. The high demand of endochondral ossification under a rapidly increasing body weight results in mechanically induced wound sites, primarily at the epiphyseal and growth plate junction. These wound sites exhibit chondronecrosis, with exposed collagen, and can intersect vasculature, thereby granting access to any and all circulating bacteria and contributing to hypoxic regions in the growth plate [2,3]. Thus, infection, inflammation, and bone attrition lead to the phenotype of FHN and, often, lameness. Several studies have found lameness to be as prevalent as 14% to 50%, and it is primarily found in larger, high-performing broilers [4,5,6]. Notably, lame birds have also been shown to respond positively to doses of anti-inflammatory and pain-reducing medication, adding to the serious animal welfare concern regarding lameness [7,8].

In addition to FHN’s deleterious effects on animal welfare and production in a commercial setting, it has become a persistent phenotype in primary breeding programs for broilers. Its persistence in breeding flocks is due to several factors. First, the diagnosis of FHN is only achieved through necropsy, resulting in the dependence on sibling data for incidence in a flock. Second, not all birds with FHN exhibit lameness. The incidence of subclinical FHN within flocks has not been fully investigated, but studies using experimental models for inducing FHN, such as wire flooring models, have found a 69.1% incidence of degrees of FHN in birds that did not develop lameness [1]. Additionally, another study found an incidence of BCO in some flocks of as high as 43 out of 100 necropsied birds [9]. Finally, the exact progression and mechanism by which FHN develops in broilers has yet to be elucidated, making non-invasive means of detection, such as circulating biomarkers, yet to be found. However, a recent study found differentially expressed cytokine and chemokine profiles in the local bone and blood of FHN-affected broilers compared to normal, healthy broilers [10]. Other researchers have utilized high-throughput transcriptomics and proteomics and identified key cartilage and bone growth as well as lipid metabolism and immune-related factors in FHS and FHN [11,12].

While protein and gene expressions are integral to cellular pathways, much of the process driving bone growth and healing as well as immune response is dependent upon the metabolic state and factors within bone. For example, the resolution of a pro-inflammatory state involves a shift from catabolic to anabolic processes within bone. Without this shift, bone attrition and inflammation remain. A main driver in this series of events is the activation or maturation of key bone cell types—most notably, osteoblasts and osteoclasts. The ratio of these two cell types is dependent on the physiological and metabolic state of bone [13,14,15]. These pathways and processes are highly dependent on and contribute to the metabolite profile in tissue. Therefore, characterizing the metabolome in pathophysiological states in broilers would be highly beneficial.

In terms of improving selection, characterizing the metabolic state of bone afflicted with FHN, particularly in birds not exhibiting lameness, could provide valuable information on how both affected and normal bone function and potential targets for nutritional interventions or the improved phenotypic description of FHN. Therefore, the present study is the first to characterize the metabolome in both normal and subclinical, FHN-affected bone in a single genetic strain of broiler breeders.

## 2. Materials and Methods

### 2.1. Birds and FHN Scoring

Broiler breeders from a single pedigree line were reared under industry standards for meat-type broilers from day 1 to day 35 of age with ad libitum access to water and feed. The feed regime throughout the growing period followed industry standards, including a starter, grower, and finisher feed. At 35 days of age, the birds were humanely euthanized and necropsied to determine the presence of FHN/BCO lesions in the proximal femora head. Femoral heads were scored macroscopically, with normal bone being free from articular cartilage cap separation as well as any necrotic lesions given a score of 0. Bone exhibiting only articular cartilage cap separation was given a score of 1, while the presence of both articular cartilage cap separation and necrotic lesions less than a pencil eraser in size was given a score of 2. Bone with cap separation and severe necrosis was given a score of 3 [1,2]. All animal handling and animal care procedures utilized followed the Cobb–Vantress animal welfare program standards that are aligned with the National Chicken Council (NCC) guidelines for broiler chickens.

### 2.2. Sample Collection and Preparation

The collection of the femur heads was only carried out for birds scoring 0 in both legs, which were considered normal (N), and those with a score greater than or equal to 2 on at least one leg and greater than or equal to 1 on the other leg, which were considered FHN-affected (F). One proximal femur head per bird, totaling 30 femur heads (N = 15), was severed at approximately the metaphysis, snap-frozen in liquid nitrogen, and stored in −80 °C.

Bone samples were ground using a mortar and pestle with liquid nitrogen to maintain the sample temperature when processing and stored at −80 °C until further analysis. Samples were sent to the Biological and Small Molecule Mass Spectrometry Core (RRID: SCR_021368). As previously described [16,17], and in brief, metabolites were extracted using 1.5 mL of extraction solvent (40:40:20 HPLC grade methanol: acetonitrile: 0.1 M final concentration of water with formic acid), prechilled at 4 °C, and incubated at −20 °C for 20 min. The samples were centrifuged at 13,300× *g* for 5 min at 4 °C before the supernatants were collected. The solvent was evaporated under a stream of nitrogen, and the metabolites were suspended in 300 μL of HPLC-grade water prior to mass spectral analysis.

### 2.3. Ultra-High-Performance Liquid Chromatography–High-Resolution Mass Spectrometry (UHPLC–HRMS) Metabolomics Analysis

UHPLC–HRMS analysis was previously published [16,17,18]. In brief, the metabolites were separated on a Dionex Ulti-Mate 3000 RS (Sunnyvale, CA, USA) by injecting a 10 μL sample on a Synergy reverse phase HydroRP 100 Å, 100 mm × 2.00 mm, 2.5 μm pore size LC column (Phenomenex, Torrance, CA, USA) that was kept at 25 °C. The global metabolomics method, which was adapted from [19], ran for 26 min with the application of a multistep gradient. Two HPLC-grade solvents were used in gradient steps to separate the analytes: solvent A (97:3 H_2_O:MeOH with 11 mM tributylamine and 15 mM acetic acid) and solvent B (100% MeOH). The gradient was performed as follows: 0 min, 0% B; 5 min, 20% B; 13 min, 55% B; 15.5 min, 95% B; 19 min, 0% B; 25 min, 0% B, with a flow rate of 200 μL/min. An electrospray ionization (ESI) source conjoined to an Exactive™ Plus Orbitrap Mass Spectrometer (Thermo Scientific, Waltham, MA, USA) was used to administer the eluent under the following parameters of aux gas: 8; sheath gas: 25; sweep gas: 3; spray voltage: 3.00 kV; and capillary temperature: 300 °C. The mass spectrometer parameters were set to resolution: 140,000; automatic gain control (AGC): 3 × 106; maximum IT time: 100; scan range: 85–1000 *m*/*z*. Raw data were obtained from the Xcalibur MS software (Thermo Electron Corp, Waltham, MA, USA) and converted to mzML format by the ProteoWizard tool MS Converter [20,21]. MAVEN [22] was used to analyze the converted data. Peaks were annotated with a maximum allowed error of 5 ppm. The area under the chromatographic curve was integrated based upon an inhouse-verified list of metabolites using the exact mass and known retention times [23]. Metabolite values were normalized based on the mass of the bone tissue extracted prior to all statistical calculations.

### 2.4. Ingenuity Pathway Analysis (IPA)

The metabolites’ fold change and *p*-value were entered into the IPA, along with their identifications, from the Human Metabolome Database, denoted as HMDB [24]; the Kyoto Encyclopedia of Genes and Genomes, denoted as KEGG [25]; and the Chemical Entities of Biological Interest, denoted as ChEBI [26]. IPA analysis was completed as previously described [17]. Briefly, the top canonical pathways and functional annotations were deduced for differentially expressed metabolites via IPA analysis. Differential expression was established using a fold change between −1.5 and 1.5 and a cutoff of an FDR-adjusted *p*-value < 0.05. Using these parameters, upstream and downstream analysis and molecular network discovery were also completed, along with the identification of biomarkers.

### 2.5. RNA Isolation, Reverse Transcription, and Real-Time Quantitative PCR

Total RNA was isolated from normal and FHN-affected bone as previously described [10]. The sequences for oligonucleotide primers for *r18s,* adenosine monophosphate (AMP)-activated protein kinases *(AMPKα1, AMPKα2*), extracellular signal-regulated kinases (*ERK1/2),* c-Jun N-terminal kinase (*JNK*)*,* and carnitine palmitoyltransferase 1 (*CPT1)* were previously published [27,28]. The primer for aquaporin 7 (*AQP7)* (forward, 5′-CCCTGAAAGGCACACATGCT-3′, and reverse, 5′-CCCATACCAATGCCCAGAAC-3′) was also used. The real-time quantitative PCR cycling conditions were 50 °C for 2 min, 95 °C for 10 min, and 40 cycles of a two-step amplification (95 °C for 15 s, followed by 58 °C for 1 min). The sequence detection system dissociation protocol was used for the melting curve analysis to omit the potential contamination of non-specific PCR products. The negative controls used as templates did not contain reverse transcription products. The 2^−∆∆CT^ method was employed to determine the relative expression of targeted genes, and healthy bone tissue was used as a calibrator [29].

### 2.6. Data Processing and Statistical Analysis

Original datasets from both groups have been submitted to the EMBL-EBI MetaboLights database (DOI: 10.1093/nar/gkz1019, PMID:31691833) with the identifier MTBLS7618 (accessed on 15 March 2023 https://www.ebi.ac.uk/metabolights/MTBLS7618). Metabolites showing differences higher or lower than 1.5-fold and a *p*-value less than 0.05 in the comparison between FHN-affected (F) and normal (N) bone were considered differentially abundant (DA). The heat maps displayed log_2_ fold changes for found metabolites and were created with R version 3.6.1. The *p*-values were calculated using Student’s *t*-test. MetaboAnalyst 5.0 [30] and the statistical package DiscriMiner in R version 3.6.1 (https://cran.r-project.org, accessed on 15 March 2023) were used for group discrimination, partial least squares discriminant analysis (PLS-DA), and variable importance in projection (VIP) scores. VIP values > 1 were considered significant due to them belonging to metabolites that contributed to group differentiation.

## 3. Results

### 3.1. Multivariate Analysis and Comparative Metabolomics Profile in FHN-Affected and Unaffected Bone

**Figure 1:**
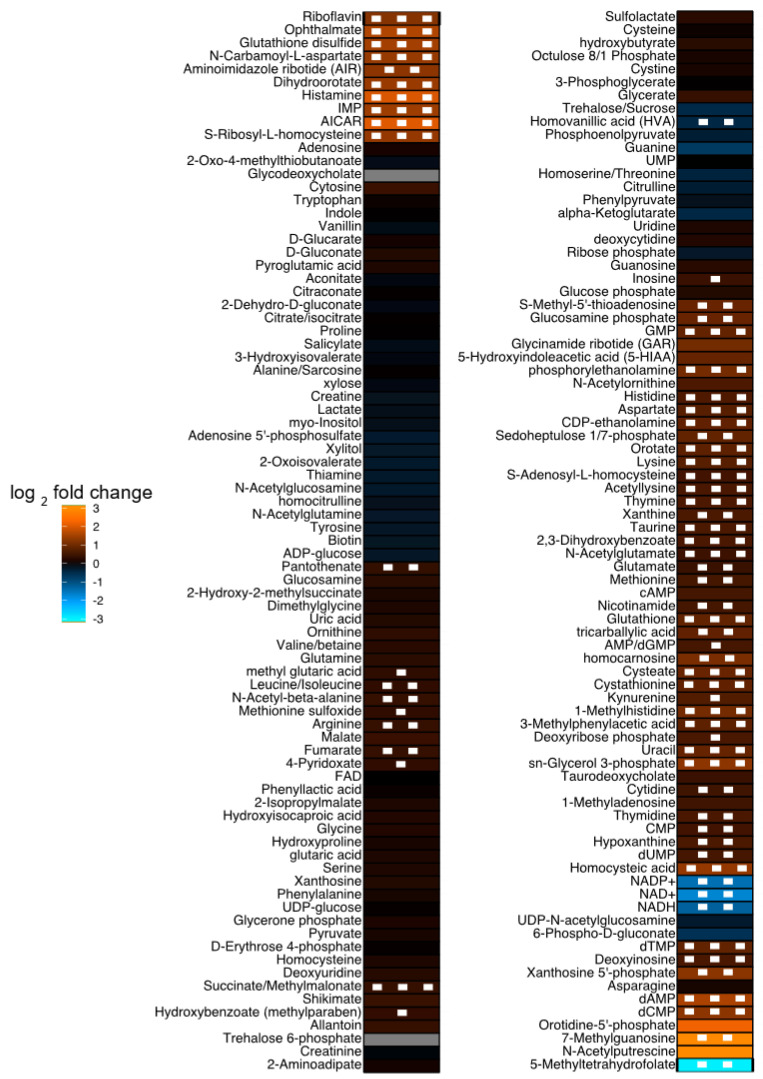
Heat map of the relative abundance of metabolites in FHN-affected compared to normal bones. A positive fold change indicates increased expression in FHN bone. Both *p*-values (indicated by squares) from a Student’s T-test and the log_2_ fold change (indicated by color) were used to build the heat map when comparing it to normal bone. Grey cells indicate an undetected metabolite. AICAR, 5-aminoimidazole-4-carboxamide1-β-D-ribofuranoside; CMP, cytidine monophosphate; dAMP, deoxyadenosine monophosphate; dCMP, deoxycytidine monophosphate; dTMP, deoxythymidine monophosphate; dUMP, deoxyuridine monophosphate; FAD, flavin adenine dinucleotide; GMP, guanosine monophosphate; IMP, inosine monophosphate; NAD+, nicotinamide adenine dinucleotide; NADH, reduced nicotinamide adenine dinucleotide; UMP, uridine monophosphate; NADP+, nicotinamide adenine dinucleotide phosphate.

**Figure 2:**
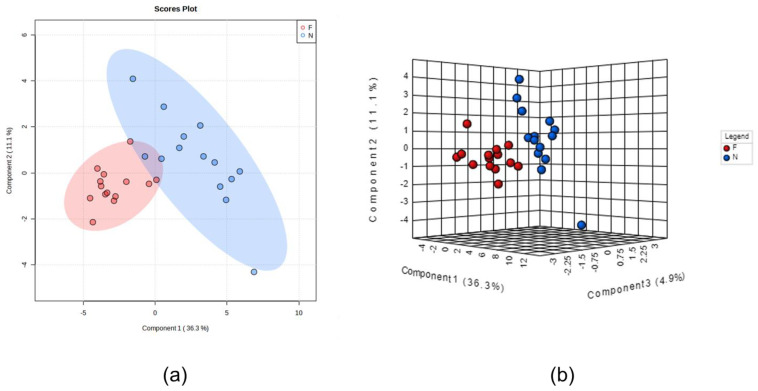
Partial least squares discriminant analysis (PLS-DA) 2D (**a**) and 3D (**b**) score plots. PLS-DA was constructed using MetaboAnalyst software and displayed different clusters when comparing the metabolite profiles in bone that is affected by FHN (F) to those in normal bone (N).

**Figure 3:**
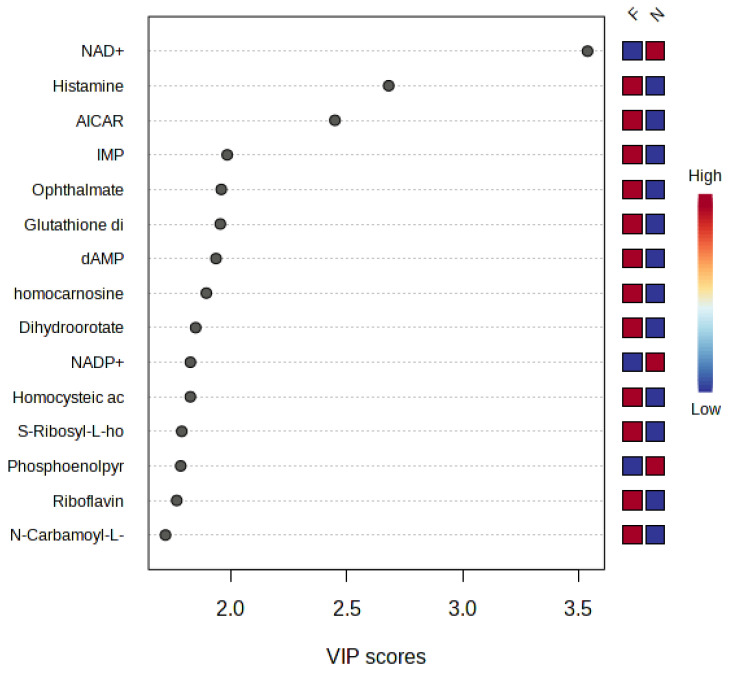
VIP scores and related cluster distribution of the top 15 DA metabolites when comparing FHN to N in PLS-DA analysis. AICAR, 5-aminoimidazole-4-carboxamide1-β-D-ribofuranoside; dAMP, deoxyadenosine monophosphate; IMP, inosine monophosphate; NAD+, nicotinamide adenine dinucleotide; NADP+, nicotinamide adenine dinucleotide phosphate.

Global metabolomics analysis identified a total of 44 differentially abundant (DA) metabolites, with 3 down-regulated and 41 upregulated in FHN-affected compared to normal bone. Figure 1 is a heat map of the identified metabolites along with the log_2_ (average relative abundance of metabolites in FHN-affected bone/the average relative abundance of metabolites in normal bone) for each metabolite detected. The PLS-DA plots, seen in Figure 2, show a clear separation of groups, indicating distinct metabolite profiles between normal and FHN-affected bone. VIP scores were assigned to each metabolite in order to assess the influence each metabolite held for the separation between the two phenotypes, with a VIP score >1 indicating significant contribution. Figure 3 shows the top 15 metabolites based on their VIP scores.

### 3.2. Metabolic Pathway and Network Analysis

**Figure 4:**
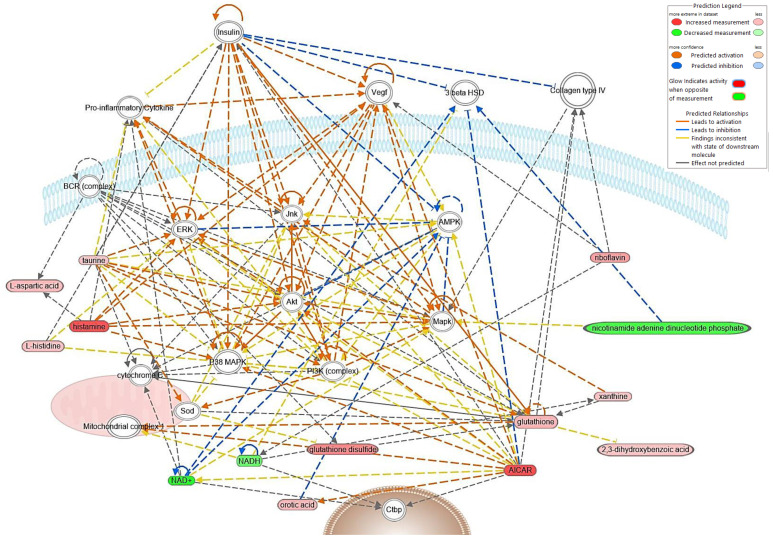
Top predicted network built with the IPA program from metabolomics data from FHN-affected bone. Metabolites in red were upregulated, while green metabolites were downregulated in FHN-affected bone. The saturation of the color indicates the level of up- or downregulation, with darker saturation indicating a more significant shift from normal bone. Orange pathway lines indicate predicted activation, and blue lines indicate predicted inhibition. Yellow pathway lines indicate the findings are inconsistent with the state of the downstream molecule, and grey lines indicate that there is not enough information to predict an effect. AICAR, 5-aminoimidazole-4-carboxamide1-β-D-ribofuranoside; Akt, serine/threonine protein kinase; AMPK, AMP-activated protein kinase; BCR, B-cell receptor; 3 beta HSD, 3β-hydroxysteroid dehydrogenase; Ctbp, C-terminal-binding protein; ERK, extracellular signal-regulated kinase; Jnk, c-Jun N-terminal kinase; Mapk, mitogen-activated protein kinase; P38 MAPK, p38 mitogen-activated protein kinase; PI3K, phosphoinositide 3-kinase; Sod, superoxide dismutase; Vegf, vascular endothelial growth factor.

Using a cut-off of an FDR-adjusted *p*-value < 0.05 and a fold-change between −1.5 and 1.5, IPA analysis identified 44 differentially abundant (DA) metabolites between FHN-affected and normal bone. A list of these metabolites, their fold change, and respective *p*-values is in Table 1. Most of the metabolites, 41, were significantly higher in FHN-affected tissue, while only 3 were significantly lower. Network predictions based on the core analysis of DA metabolites in IPA found seven networks. The top three networks involved 15, 12, and 11 focus molecules and were related to amino acid metabolism, molecular transport, cellular growth and proliferation, energy production, organismal development, lipid metabolism, and small molecule biochemistry. A compilation of these three networks is summarized in Figure 4. The predicted upstream and downstream regulator complexes and molecules based on DA metabolites included collagen type IV, 3β-hydroxysteroid dehydrogenase (3β HSD), AMP-activated protein kinase (AMPK), insulin, vascular endothelial growth factor (Vegf), P38 mitogen-activated protein kinase (P38 MAPK), Akt protein kinase, extracellular signal-regulated kinase (ERK), and c-Jun N-terminal kinase (Jnk).

**Figure 5:**
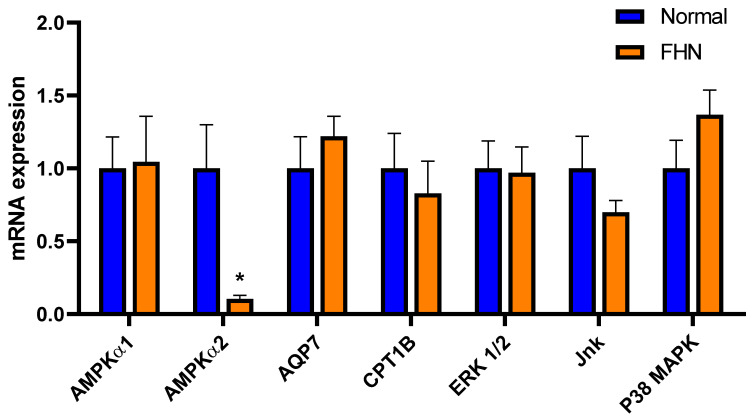
Assessment of IPA-predicted genes by real-time qPCR. The relative expression of target genes was measured by the 2^-ΔΔCt^ method. Data are means ± SEM. * indicates statistical significance of a *p*-value < 0.05. AMPK, AMP-activated protein kinase; AQP7, aquaporin 7; CPT1B, carnitine palmitoyltransferase 1B; ERK, extracellular signal-regulated kinase; Jnk, c-Jun N-terminal kinase; P38 MAPK, p38 mitogen-activated protein kinase.

The top 5 canonical pathways generated based on DA metabolites were ascorbate recycling, purine nucleotides degradation II, purine nucleotides de novo biosynthesis II, urate biosynthesis/inosine 5′-phosphate degradation, and guanosine nucleotides degradation III (Table 2). The top altered diseases and functions in FHN-affected bone compared to normal bone were cancer, organismal injury and abnormalities, dermatological disease and conditions, hematological disease, and gastrointestinal disease (Table 3). Cancer, organismal injury and abnormalities, and gastrointestinal disease had the most molecules associated with them compared to other diseases and functions. Metabolomic analysis using IPA found the molecular functions associated with the unique metabolite profile in FHN-affected bone to be lipid metabolism, small molecule biochemistry, cellular development, cellular growth and proliferation, and nucleic acid metabolism (Table 4). The top upstream regulators according to DA metabolites were found with IPA and included ibrutinib, taxia-telangiesctasia mutated (ATM), carnitine palmitoyltransferase 1B (CPT1B), cluster of Differentiation 40 (CD40), CD274, D-glucose, choline kinase alpha (CHKA), and aquaporin 7 (AQP7) (Table 5). The predicted upregulation of AMPK in IPA was confirmed via real-time PCR for *AMPKα2* (Figure 5). However, *AMPKα1* expression was not significantly affected. Additionally, IPA’s predicted differential expression of AQP7, CPT1B, ERK, Jnk, and P38 MAPK was not confirmed via real-time PCR (Figure 5).

## 4. Discussion

FHN, and its associated lameness, has remained a major concern for animal welfare and poultry production throughout years of improved genetic selection and broiler performance. The inability to reliably detect all FHN through gate scoring, the lack of a mechanistic understanding, and the absence of confirmed progression of the disease have made FHN one of the most challenging physiological obstacles in poultry. The phenotype of FHN is clear: bacterial infection by highly pathogenic, opportunistic bacteria of weak bone in often high-performing broilers resulting in chondronecrosis and osteomyelitis [31,32]. The mechanism by which bacteria induce FHN has also begun to be investigated, with molecular pathways such as autophagy and mitochondrial function being implicated [33,34]. An understanding of the systemic effects of FHN as well as a foundation for non-invasive biomarkers have also been established via the evaluation of the cytokine and chemokine profiles in the blood and bone of normal and FHN-affected broilers [10]. The severity of disrupted cellular functions and overall bone homeostasis is becoming clearer as high-throughput omics studies are employed. Recent studies of the transcriptome and proteome revealed major alterations to bone development, cellular proliferation, and the immune response from normal bone [11,12,35]. Here, we found the differences between normal and FHN-affected bone to exist within the metabolome of these tissues as well. High-throughput metabolomics analysis and PLS-DA scores plots revealed distinct metabolite profiles for unaffected and FHN-affected bone. These DA metabolites revealed major metabolic pathways and molecular categories to be implicated in FHN, such as energy production, lipid metabolism, and cellular growth and proliferation.

Specifically, a clear drop in NAD+, NADP, and NADH was found in FHN-affected bone, with NAD+ being the top contributing molecule to cluster separation based on VIP scores. Not only is NAD+ and its constituents essential for energy production, but it also has been implicated in regulating several cellular functions. The implications for such a significant reduction in NAD+ in FHN-affected bone could contribute to the widespread deterioration of bone integrity starting at the early stages of bone cell lineage and via the modulation of cellular energy production. Notably, with aging, decreased levels of NAD+ have been shown to contribute to a loss of bone mass and a reduction in osteoprogenitors [36,37]. Additionally, NAD+ reduction has been shown to alter the drive of the adipo-osteogenic lineage commitment of bone marrow mesenchymal stem cells away from osteogenesis, potentially through its relationship with oxidative phosphorylation (OxPhos) [38]. NAD+ levels have also been associated with the intracellular NAMPT-NAD+-SIRT1 cascade necessary for the improved repair of vasculature post-ischemia [39]. Network analysis showed that NAD+, NADP, and NADH contribute to the predicted inhibition of AMPK and 3β HSD. Indeed, AMPKα2 mRNA expression was significantly down-regulated in FHN-affected compared to normal bone. The involvement of these metabolites in the inhibition of AMPK could also relate to bone loss and the balance of bone resorption to bone formation. In addition to AMPK’s notoriety as the master energy sensor and regulator of energy balance, AMPK’s gate keeping of energy homeostasis has made it a key player in bone cell generation and overall osteogenesis. Several studies have shown different means by which AMPK activation relates to reduced bone loss and the suppression of bone resorption through the modulation of osteoclast generation [40,41,42,43,44]. The enzyme 3β-HSD is responsible for conversion of dehydroepiandrosterone (DHEA) into δ4-androstenedione [45]. This process has been found to occur in osteoblast-like cells and has been implicated in the hormonal regulation of bone remodeling in both men and women [46,47]. This pathway has also been associated with the relationship between hormonal signaling and immune activation, particularly with respect to Th1/Th2 cells [48]. The inhibition of this complex could follow the trend of AMPK inhibition with a shift in bone remodeling into the attrition seen in FHN. This, in combination with a shift in the cell lineage away from bone forming cell types, could confound the already necrotic bone conditions seen in FHN. Additionally, the alteration of several key energy production molecules indicates a decrease in energy production and increased catabolism within FHN. However, what has yet to be elucidated is whether the drop in NAD+ and the predicted subsequent effects are the result of or the cause of the FHN phenotype.

AICAR was the third most significantly differentially expressed metabolite in FHN-affected bone based on the VIP score, with an increased fold change of 4.166 (*p* = 0.00014). Within cells, AICAR is converted to the AMP analogue ZMP via intracellular adenosine kinase, mimicking cellular metabolic stress [49]. In mice lacking AMPK β1/β2 subunits, AICAR administration led to decreased bone mass and increased osteoclast formation [50]. As seen in the network analysis, the increased levels of AICAR in FHN bone contradict the predicted inhibition of AMPK. However, the AICAR effects on osteoclasts were shown to be independent of AMPK. Further research is needed into the potential mechanism by which AICAR contributes to FHN pathophysiology, especially in relation to energy expenditure and AMPK activation.

Histamine was found to be significantly abundant (fold change of 4.023, *p* = 0.00077) in FHN-affected bone compared to normal bone and a significant contributor to the differences in metabolic profiles based on VIP scores. An endogenous amine with roles in allergic reactions and gastric acid production, histamine, has also been implicated in bone remodeling, with excess histamine release in mastocytosis and some allergic diseases leading to the development of osteoporosis [51,52]. Indeed, histamine has been shown to increase bone resorption through its effects on osteoclasts and osteoclast precursors, and it increases RANKL expression in osteoblasts [52,53]. Metabolites in FHN-affected bone have influence over the ratio of bone-forming to bone-absorbing cells, with a favor towards bone absorption that could contribute to the lack of healing seen in FHN.

The top molecular function found that is related to FHN DA metabolites was lipid metabolism, indicating another means of bone cell lineage disruption and the loss of bone integrity. The significant drop in NADP and NADH as well as increased glutathione were some of the 12 contributing molecules that led to the prediction of lipid metabolism being involved in FHN. Specifically, both the lipolysis and hydrolysis of the phospholipid were predicted to be activated, and the conversion of the lipid was predicted to be inhibited. Fat infiltration in bone has been characterized in age-related bone loss and is a hallmark of decreased bone integrity and density [54]. Indeed, increased lipid metabolism can contribute to a systemic pro-inflammatory state, associated with FHN [10]. Lipid metabolism has been directly related to osteoporosis, characterized by decreased bone mass and deteriorating microstructure within the bone. An osteoporotic mouse model showed that glucocorticoid stimulation led to adipocyte aggregation and both increased cholesterol and decreased bone mineral density levels [55,56]. In FHN, lipid metabolism was also predicted to be involved based on transcriptomic analysis and serum chemistry analysis in femur head separation and was found to be altered in broilers with spontaneous FHN [11,57,58]. Additionally, the accumulation of lipid droplets in the liver of broilers with spontaneous FHN was also seen, further implicating lipid dysmetabolism with FHN pathology, although the exact relationship has yet to be determined [58]. The proteomics of the plasma from FHN-affected broilers also revealed an alteration of key lipid profiles in FHN, including elevated apolipoprotein A1 [59].

The top canonical pathway associated with DA metabolites revealed ascorbate recycling, predicted to be significantly inhibited (z-score −0.447). Not only does ascorbate recycling help maintain redox homeostasis, but its inhibition in bone could also influence bone matrix formation [60,61]. Markedly, ascorbate influences osteoblast differentiation and is a cofactor for collagen synthesis [62]. The inhibition of ascorbate recycling could contribute to reactive oxidative stress, implied in other studies of FHN, and further affect the deteriorating collagen microstructure in FHN, particularly as the bone continues to undergo endochondral ossification. Another canonical pathway found in IPA analysis was purine nucleotides degradation. Purines have long been documented as extracellular signaling molecules [63]. Purines and other nucleotides have been shown to signal through P2 receptors in regulating bone and cartilage metabolism via the activation of osteoclasts and the inhibition of osteoblasts [64,65]. Indeed, adenosine derivatives can directly affect bone cells via membranal receptors, modulating bone growth and healing [65]. While it was not determined whether this or other canonical pathways were activated or inhibited based on the z-score, their predicted involvement in addition to ascorbate recycling inhibition relates directly to bone homeostasis and the state of bone cell populations under FHN conditions.

The analysis of the FHN-affected bone metabolite profile with IPA found several upstream regulators including the predicted activation of CD40 and aquaporin 7 (AQP7) and the inhibition of ATM and CPT1B. CD40 is a member of the tumor necrosis factor family of receptors which is presented by B cells and helps mediate adaptive immunity as well as other immune-related and cellular functions [66]. In bone, single nucleotide polymorphisms of CD40 have been associated with decreased bone mineral density [67]. CD40 expression was shown to be induced by bacterial infection in human and mouse osteoblast cells. A recent study with CRISPR-Cas9 CD40 knock-out epithelial cells showed that bacterial species such as *Staphylococcus aureus* interact with CD40 as an integral part of their pathology, specifically in inducing chemokine production [68]. The bacterial infection in FHN-affected bone could be activating CD40 as a regulator of further downstream immune-related responses. The appearance of AQP7 as an upstream regulator is not unexpected given the predicted involvement of lipid metabolism. AQP7 is the main transporter for glycerol in adipose tissue and is known for its implications in energy metabolism and body fluid homeostasis [69,70]. Although the exact role of AQP7 in bone is still unclear, it is possible that AQP7 regulates the function (migration, differentiation, etc.) of mesenchymal cells (MSCs), the progenitor of osteoblasts [71]. In fact, it has been shown that AQP expression is associated with high concentrations of collagen type II, aggrecan, and lubricin, thus demonstrating the relationship between these channel proteins and chondrocyte- ECM adhesion and migration [72]. While real-time PCR analysis of *AQP7* and *CPT1B*, as well as *ERK1/2*, *Jnk*, and *P38 MAPK*, did not confirm differential expression in FHN-affected bone, further research into the protein expression of these factors could provide more insight into their state in bone.

In conclusion, this is the first study to use high-throughput metabolomics to assess differences between normal and FHN-affected bone from a single genetic strain of broiler breeders without clinical lameness. Our results show a distinct metabolite profile in FHN-affected bone with implications for both energy production and bone integrity via the modulation of metabolites, with direct relationships to bone cell differentiation, proliferation, and function. In addition to providing new insights into potential mechanisms of FHN pathology, the differentially abundant metabolites found could be further studied to determine their legitimacy as potential biomarkers or therapeutic or nutritional targets—particularly, in addressing the state of bone cells and the balance of resorption and formation within FHN-affected bone necessary for healing and new bone growth, rather than attrition and inflammation. Further research is needed into whether these pathways and metabolites serve as a cause of or consequence of FHN and whether targeting them in nutritional, genetic, and/or managerial interventions proves beneficial.

## Figures and Tables

**Table 1:** Differentially abundant metabolites in FHN-affected bone.

HMDB ID	Metabolite Name	Fold Change	*p*-Value
**Increased abundance**			
HMDB0001517	AICAR	4.166	0.00014
HMDB0000870	Histamine	4.023	0.00077
HMDB0005765	Ophthalmate	3.103	0.00062
HMDB0000905	dAMP	2.981	0.00033
HMDB0003337	Glutathione disulfide	2.841	0.00002
HMDB0015536	IMP	2.764	0.00083
HMDB0003349	Dihydroorotate	2.652	0.00086
HMDB0000828	*N*-Carbamoyl-l-aspartate	2.621	0.00031
HMDB0000676	*S*-Ribosyl-l-homocysteine	2.590	0.00167
HMDB0002205	Homocysteic acid	2.558	0.00010
HMDB0000126	*sn*-Glycerol-3-phosphate	2.450	0.00182
HMDB0001202	dCMP	2.446	0.00110
HMDB0001235	Aminoimidazole ribotide	2.438	0.01686
HMDB0001554	Xanthosine-5′-phosphate	2.402	0.01264
HMDB0000244	Riboflavin	2.340	0.00031
HMDB0000745	Homocarnosine	2.163	0.03150
HMDB0000224	Phosphorylethanolamine	2.108	0.00485
HMDB0000001	1-Methylhistidine	2.107	0.00337
HMDB0002757	Cysteate	1.909	0.00011
HMDB0001173	*S*-Methyl-5′-thioadenosine	1.904	0.01450
HMDB0001254	Glucosamine phosphate	1.903	0.01654
HMDB0000300	Uracil	1.866	0.00735
HMDB0001397	GMP	1.844	0.00194
HMDB0060509	Sedoheptulose-1/7-phosphate	1.830	0.03038
HMDB0000125	Glutathione	1.826	0.00034
HMDB0001227	dTMP	1.826	0.00054
HMDB0001564	CDP-ethanolamine	1.818	0.00109
HMDB0031193	Tricarballylic acid	1.805	0.02514
HMDB0000099	Cystathionine	1.803	0.00095
HMDB0000226	Orotate	1.785	0.00104
HMDB0002222	3-Methylphenylacetic acid	1.784	0.00025
HMDB0003405	Lysine	1.696	0.00116
HMDB0000191	Aspartate	1.678	0.00020
HMDB0000177	Histidine	1.594	0.00093
HMDB0000262	Thymine	1.562	0.00219
HMDB0000251	Taurine	1.545	0.00111
HMDB0000292	Xanthine	1.540	0.02493
HMDB0000157	Hypoxanthine	1.534	0.01077
HMDB0000397	2,3-Dihydroxybenzoate	1.524	0.00089
HMDB0000273	Thymidine	1.514	0.03461
HMDB0000206	Acetyllysine	1.514	0.00196
HMDB0000071	Deoxyinosine	1.511	0.00786
HMDB0001409	dUMP	1.471	0.03402
HMDB0000696	Methionine	1.463	0.02533
HMDB0001406	Nicotinamide	1.459	0.02024
HMDB0000095	CMP	1.452	0.01468
HMDB0000939	I-Adenosyl-l-homocysteine	1.449	0.00441
HMDB0000089	Cytidine	1.432	0.01883
HMDB0001138	*N*-Acetylglutamate	1.395	0.00092
HMDB0060475	Glutamate	1.344	0.01180
HMDB0000202	Succinate/Methylmalonate	1.325	0.00358
HMDB0000134	Fumarate	1.324	0.02559
HMDB0000517	Arginine	1.314	0.03810
HMDB0061880	N-Acetyl-beta-alanine	1.297	0.01459
HMDB0000210	Pantothenate	1.290	0.03354
HMDB0028932	Leucine/Isoleucine	1.255	0.03115
HMDB0000118	Homovanillic acid	0.721	0.02502
**Decreased abundance**			
HMDB0001487	NADH	0.417	0.04303
HMDB0000217	NADP+	0.360	0.02809
HMDB0000902	NAD+	0.300	0.01608

AICAR, 5-aminoimidazole-4-carboxamide1-β-D-ribofuranoside; CMP, cytidine monophosphate; dAMP, deoxyadenosine monophosphate; dCMP, deoxycytidine monophosphate; dTMP, deoxythymidine monophosphate; dUMP, deoxyuridine monophosphate; FAD, flavin adenine dinucleotide; GMP, guanosine monophosphate; IMP, inosine monophosphate; NAD+, nicotinamide adenine dinucleotide; NADH, reduced nicotinamide adenine dinucleotide; UMP, uridine monophosphate; NADP+, nicotinamide adenine dinucleotide phosphate.

**Table 2:** Top canonical pathways enriched by metabolite alterations detected in FHN-affected bone.

Canonical Pathway	Molecules	−log (*p*-Value)	Ratio
Ascorbate Recycling (Cytosolic)	glutathione, glutathione disulfide, NAD+, NADH, NADP+	6.99	0.625
Purine Nucleotides Degradation II (Aerobic)	GMP, hypoxanthine, NAD+, NADH, xanthine, xanthosine monophosphate	6.47	0.353
Purine Nucleotides De Novo Biosynthesis II	AICAR, aminoimidazole ribotide, GMP, l-aspartic acid, NAD+, NADH, xanthosine monophosphate	6.12	0.233
Urate Biosynthesis/Inosine 5′-phosphate Degradation	NAD+, NADH, xanthine, xanthosine monophosphate	4.88	0.444
Guanosine Nucleotides Degradation III	GMP, NAD+, NADH, xanthine	4.66	0.4

**Table 3:** Top diseases associated with the metabolite profile in FHN-affected bone.

Diseases and Functions	*p*-Value	# Molecules *
Cancer	4.94 × 10^−2^–8.20 × ^10−6^	19
Organismal Injury and Abnormalities	4.94 × 10^−2^–8.20 × 10^−6^	23
Dermatological Disease and Conditions	3.78 × 10^−2^–1.73 × 10^−4^	6
Hematological Disease	1.36 × 10^−2^–4.95 × 10^−4^	4
Gastrointestinal Disease	3.78 × 10^−2^–7.36 × 10^−4^	14

# means number which is the explained. * Number of molecules with each network. IPA generated diseases and functions based on the association with this database and our input data. The *p*-value was calculated using Fisher’s exact Test by IPA.

**Table 4:** Molecular and cellular functions associated with the metabolomic profile in FHN-affected bone.

Molecular and Cellular Functions	*p*-Value	# Molecules *
Lipid Metabolism	4.00 × 10^−2^–1.23 × 10^−4^	12
Small Molecule Biochemistry	4.93 × 10^−2^–1.23 × 10^−4^	17
Cellular Development	4.66 × 10^−2^–2.12 × 10^−4^	15
Cellular Growth and Proliferation	4.66 × 10^−2^–2.12 × 10^−4^	14
Nucleic Acid Metabolism	4.14 × 10^−2^–3.54 × 10^−4^	12

# means number which is the explained. * Number of molecules with each function. The molecular functions were produced with IPA database association and our input data. The *p*-values were calculated using Fisher’s exact Test by IPA.

**Table 5:** Upstream regulators and their relative z-score in relation to metabolites in FHN-affected bone.

Upstream Regulators *	*p*-Value	Z Score
ATM	6.20 × 10^−7^	−1.732
CPT1B	8.78 × 10^−6^	−1.633
CD40	1.20 × 10^−4^	2.236
CD274	6.16 × 10^−7^	1
CHKA	9.55 × 10^−7^	−0.577
AQP7	1.86 × 10^−4^	1.566

* ATM, ataxia-telangiesctasia mutated; AQP7, aquporin 7; CD, cluster of differentiation; CHKA, choline kinase alpha; CPT1B, carnitine palmitoyltransferase 1B.

## Data Availability

Data are available in the EMBL-EBI MetaboLights database (DOI: 10.1093/nar/gkz1019, PMID:31691833) with the identifier MTBLS7618 (accessed on 15 March 2023 https://www.ebi.ac.uk/metabolights/MTBLS7618).
